# Effects of concentrate feeding sequence on VFA production, and cecal microbiota of Dezhou donkeys by metagenomic technology

**DOI:** 10.3389/fvets.2024.1401980

**Published:** 2024-06-04

**Authors:** Changyun Cai, Lan Xie, Jingya Xing, Ting Lu, Xingzhen Qi, Lanjie Li, Xue Chen, Muhammad Faheem Akhtar, Yaqian Jin, Guiqin Liu

**Affiliations:** ^1^College of Agronomy and Agricultural Engineering, Liaocheng University, Shandong Engineering Technology Research Center for Efficient Breeding and Ecological Feeding of BlackDonkey, Shandong Donkey Industry Technology Collaborative Innovation Center, Liaocheng, China; ^2^College of Animal Science, Qingdao Agricultural University, Qingdao, China; ^3^Office of International Programs, Liaocheng University, Liaocheng, China

**Keywords:** Dezhou donkeys, Cecal microbiota, metagenomic technology, concentrate feeding sequence, VFA

## Abstract

Microorganisms residing in the cecum of donkeys are crucial for physiological processes, nutrient metabolism, and immune function. Feeding methods can affect the dynamic balance of animal gut microbiota, thereby affecting indicators such as volatile fatty acids. This study explores suitable feeding methods to promote actual production by changing the feeding order of concentrate. Fifteen Dezhou donkeys with similar age and weight profiles were randomly divided into three groups with the concentrate feeding sequence: fiber-to-concentrate (FC), concentrate-to-fiber (*CF*), and total mixed ration (TMR). The experiment spanned a duration of 82 days. The analyses conducted were primarily aimed at determining the effects of feeding on gut microbes, primarily using metagenomic sequencing techniques. The experimental findings revealed that the levels of valeric acid were notably higher in the *CF* and TMR groups compared to the FC group (*p* < 0.05). These results suggest that the feeding sequence exerts a certain impact on the microbial composition within the cecum of Dezhou donkeys. At the phylum level, the predominant microbiota consisted of Firmicutes and Bacteroidetes, with the *CF* group displaying a higher relative abundance of Firmicutes compared to both the FC and TMR groups. At the genus level, *Prevotella*, *Bacteroides*, and *Fibrobacter* were the dominant bacterial genera identified in cecum. The functional gene annotation analysis indicated a significantly lower abundance of *lacZ* (K01190), *Por/nifJ* (K03737), and *ppdK* (K01006) genes in *CF* group relative to the FC and TMR groups (*p* < 0.05), highlighting their roles in galactose metabolism and glycolysis, respectively. Moreover, the *CF* group exhibited a higher concentration of antibiotic resistance genes (*tetO* and *tet44*) in the gut microbiota compared to the TMR and FC groups (*p* < 0.05), underscoring the presence of numerous antibiotic resistance genes within the phyla Bacteroidetes, Firmicutes, and Proteobacteria. In conclusion, different precision feed sequences significantly impact the levels of volatile fatty acids in Dezhou fattening donkeys, modify the composition and functional genes of the cecal microbiota, and elucidate the microbial mechanisms influenced by the feeding sequence on the growth and metabolism. These insights are anticipated to provide a foundation for the rational design of precision feed sequences in practical agricultural settings.

## 1 Introduction

Donkeys classified within the Mammalia class and the order Perissodactyla, belong to the family Equidae, specifically the genus Equus. In China, there are currently 24 recognized indigenous donkey breeds, along with Mongolian wild donkey, and the Tibetan donkey (1). Notably, the Dezhou donkey originating from the LuBei region of Shandong Province along the Bohai Sea and predominantly found in Wudi County (2), is distinguished as one of the top five donkey breeds in China (3). As societal preferences evolve toward diverse and health-conscious dietary options, donkey-derived products such as ejiao, donkey meat, and milk have gained popularity (4, 5). Consequently, the primary role of donkeys has shifted from labor to food production, fostering the development of a market driven by specialized branding enterprises (6). In recent years, the donkey-raising industry has emerged as a novel sector in China. It is imperative to document the historical significance of donkeys, develop and refine the technical frameworks within the industry, and actively engage in the conservation and introduction of superior breeds (7). Additionally, there is a need to develop an integrated brand industry that encompasses production, processing, services, and scientific research (8).

Donkeys are herbivorous animals, and traditional breeding mainly relies on green and fiber feed, supplemented by a small amount of concentrate feed (9). The use of this feeding method has drawbacks such as reduced feed intake and low efficiency in utilizing feed resources (10). Forage is the foundation of large-scale breeding of donkeys. With the transformation of breeding methods and target products, the feeding methods and nutritional requirements of donkeys are different from traditional breeding, and the requirements for forage and diet will also change accordingly. The survey mainly focused on large-scale donkey farms in Shandong Province, and conducted investigations, statistics, and sampling analysis on the types, sources, and feeding conditions of forage. The survey results show that corn silage, peanut seedlings, and soybean straw are the most important forage in large-scale donkey farms. At the same time, 95% of donkey farms added concentrate feed to their diets, and only 4 donkey farms stopped adding concentrate feed due to feeding whole plant corn silage (11). The rapid development of large-scale breeding of donkeys does not match the supporting breeding and management techniques. Currently, most of them draw on the experience of large-scale breeding of cattle and sheep, but in terms of behavioral habits and digestive physiology, donkeys have significant differences from cattle and sheep. Therefore, it is necessary to explore suitable dietary formulations and feeding methods as soon as possible.

There are few studies on the nutritional requirements of donkeys at China and abroad, and the dietary needs of young horses are usually referred to when feeding donkeys (12). Research has demonstrated that employing a specific feeding sequence yields better weight gain and reduces the incidence of digestive tract diseases compared to unsequenced mixed feeding (13). Administering concentrates after fiber feed, due to the palatability of concentrates, stimulates a positive appetite reflex, enhances gastrointestinal motility, and promotes digestion (14). Overfeeding concentrates, however, reduces the intake of fiber feed (15). Conversely, when fiber feed precedes concentrate, the intake of concentrates is diminished due to the satiety effects of the roughage (16). Total Mixed Rations (TMR) are extensively utilized in ruminants such as dairy cows and represent a key technology for achieving balanced feeding in these species (17). Experimental data show that using the TMR feeding method for large-scale fattening can improve weight gain rate and shorten fattening time (18).

As a monogastric herbivore, donkeys possess a well-developed cecum harboring diverse microbial populations that play a pivotal role in the host’s physiological, metabolic, nutritional, and immunological functions (19). Evolved as grazing animals, equines exhibit rapid gastric transit, vigorous enzymatic digestion in the small intestine, and prolonged microbial fermentation in the large intestine (20). The gastrointestinal microbiota is crucial for the nutrition and health of monogastric herbivores, including donkeys (21). Advanced sequencing techniques, such as 16S rRNA gene sequencing and metagenomics, have been employed to characterize the digestive system in terms of microbial composition and function. These findings provide essential baseline data on the gastrointestinal microbiota, which aids in assessing the growth and development of donkeys and offers insights for further research into their digestive systems, nutrition, and potential development of microbial supplements (22).

In the study of microbial diversity and community characteristics, compared with high-throughput sequencing technology, metagenomic sequencing technology can better mine microbial genomic information and further study microbial flora’s biodiversity and community structure (23). Therefore, based on metagenomic technology, this study analyzed the effects of different concentrate feeding sequences on the growth and development, nutritional requirements and, cecal microbial diversity of fattening donkeys. The promotion of large-scale breeding provides a theoretical basis for selecting different concentrated feeding sequences of fattening donkeys.

## 2 Materials and methods

### 2.1 Animals and groups

The experimental animals were provided by Donge-e-Jiao Co Ltd. Fifteen healthy Dezhou fattening male donkeys aged 2 years ±3 months and weighing (215 ± 10) kg were randomly divided into 3 groups with 5 donkeys in each group. The groups were divided into the FC group (feeding sequence was fiber first followed by concentrate), *CF* group (feeding sequence was concentrate first followed by fiber), and TMR group (feeding sequence was TMR).

### 2.2 Experimental diet

All experimental donkeys were raised in a single pen. Throughout the testing period, the environment in the donkey house was kept clean, and the utensils inside the house were regularly disinfected. The experiment lasted for 82 days, with 7 days of pre-feeding and 75 days of formal feeding. During the experiment, it was fed twice a day (07:00 and 17:00). The feed rates of concentrate feed and beanstalk were 1.3 and 2.0% of body weight, respectively. During the experiment, the feed rates were weighed and adjusted every 30 days. The *CF* group was fed with fiber feed after being fed with concentrate feed for 0.5 h, while the FC group was fed with concentrate feed after being fed with fiber feed for 0.5 h. The TMR group mixed the fiber feed, concentrate feed, and water in a certain proportion to form TMR and fed it freely with drinking water. The composition and nutritional level of concentrate feed are summarized in Table 1. The crude feed was beanstalk, and its nutritional level as mentioned in Table 2.

**Table 1:** Nutrient levels of beanstalk (air-dry basis)/%.

Nutrient levels	Contents
CP	5.31
EE	0.39
*CF*	43.39
ADF	53.53
NDF	64.20
DE/(MJ/kg)	2.65

CP, crude protein; EE, crude extract; CF, crude fiber; NDF, neutraldetergent fiber; ADF, acid detergent fiber; DE, digestibility energy. DE was a calculated value, while the others were measured values.

**Table 2:** Feed composition and nutrient composition /%.

Items	Contents
Ingredients	
Corn	53.60
Soybean meal	19.40
Peanut meal	12.00
Corn germ	6.00
Stone power	4.00
Premix^1^	5.00
Total	100.00
Nutrient level^2^	
CP	20.18
*CF*	4.64
EE	1.68
ADF	6.90
NDF	12.73
DE/(MJ/kg)	7.95

^1^Premix provides per kg of feed:V_E_ 50 mg, V_A_ 20,000 IU, V_K_ 2.5 mg, V_D_ 3,500 mg, V_B1_ 2.5 mg, V_B2_ 8.0 mg, V_B3_ 25 mg, V_B5_ 32 mg, V_B6_ 0.5 mg, V_B12_ 50 ug, Folic Acid 0.5 mg, biotin 90 ug, Fe 200 mg, Mn 50 mg, Zn 220 mg, Cu 30 mg, Se 0.45 mg, I 2.0 mg; ^2^CP, crude protein; EE, crude extract; CF, crude fiber; NDF, neutral detergent fiber; ADF, acid detergent fiber; DE, digestibility energy. DE was a calculated value, while the others were measured values.

### 2.3 Sample collection

Following the feeding trial, all donkeys were euthanized according to standard procedures. Prior to sampling, necessary preparations were made, which included assembling relevant experimental materials, such as sterile scissors, sterile tweezers, sterile enzyme-free freeze-storage tubes, gloves, markers for liquid nitrogen tanks, etc. Subsequently, the cecal intestinal contents were aseptically collected from the experimental donkeys. Samples were then transferred into at least three sterile, enzyme-free freeze-storage tubes, securely sealed, and immediately placed into liquid nitrogen for transport. These samples were subsequently stored at-80°C until further analysis. The cecal contents are taken from the apex of the cecum (C).

### 2.4 Determination index VFA

For the analysis of volatile fatty acids (VFAs), approximately 50 mg of the cecal content samples were placed into a 2 mL centrifuge tube. To this, 50 μL of 15% phosphoric acid, 100 μL of 125 μg/mL internal standard solution (4-methylvaleric acid), and 400 μL of ether were added. The mixture was homogenized by vigorous shaking for 1 min. The samples were then centrifuged at 12,000 × g at 4°C for 10 min. The supernatant was carefully transferred into a vial in preparation for gas chromatography–mass spectrometry (GC–MS) analysis.

### 2.5 Process of sequencing and bioinformation analysis

#### 2.5.1 Extraction, purification, and library construction of sample DNA

**Figure 1:**
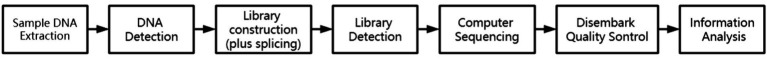
Experimental flow chart.

Initially, all samples underwent a preliminary testing phase. DNA samples that met the quality criteria were subsequently fragmented into segments using an ultrasonic crusher. The library construction involved several key steps: terminal repair, A-tail addition, sequencing joint expansion, purification, PCR amplification, and additional cleanup steps. Upon completion of these steps, the libraries were initially quantified. Following dilution, the libraries underwent a series of evaluations to confirm they met predefined standards. The effective concentration of the libraries was then precisely quantified using quantitative real-time PCR to ensure the integrity and quality of the libraries. DNA extraction and purification are assisted by Beijing NuoHeZhiYuan Biological Information Technology Co Ltd. The specific sequencing process of library construction is illustrated in Figure 1.

#### 2.5.2 Bio information analysis process

**Figure 2:**
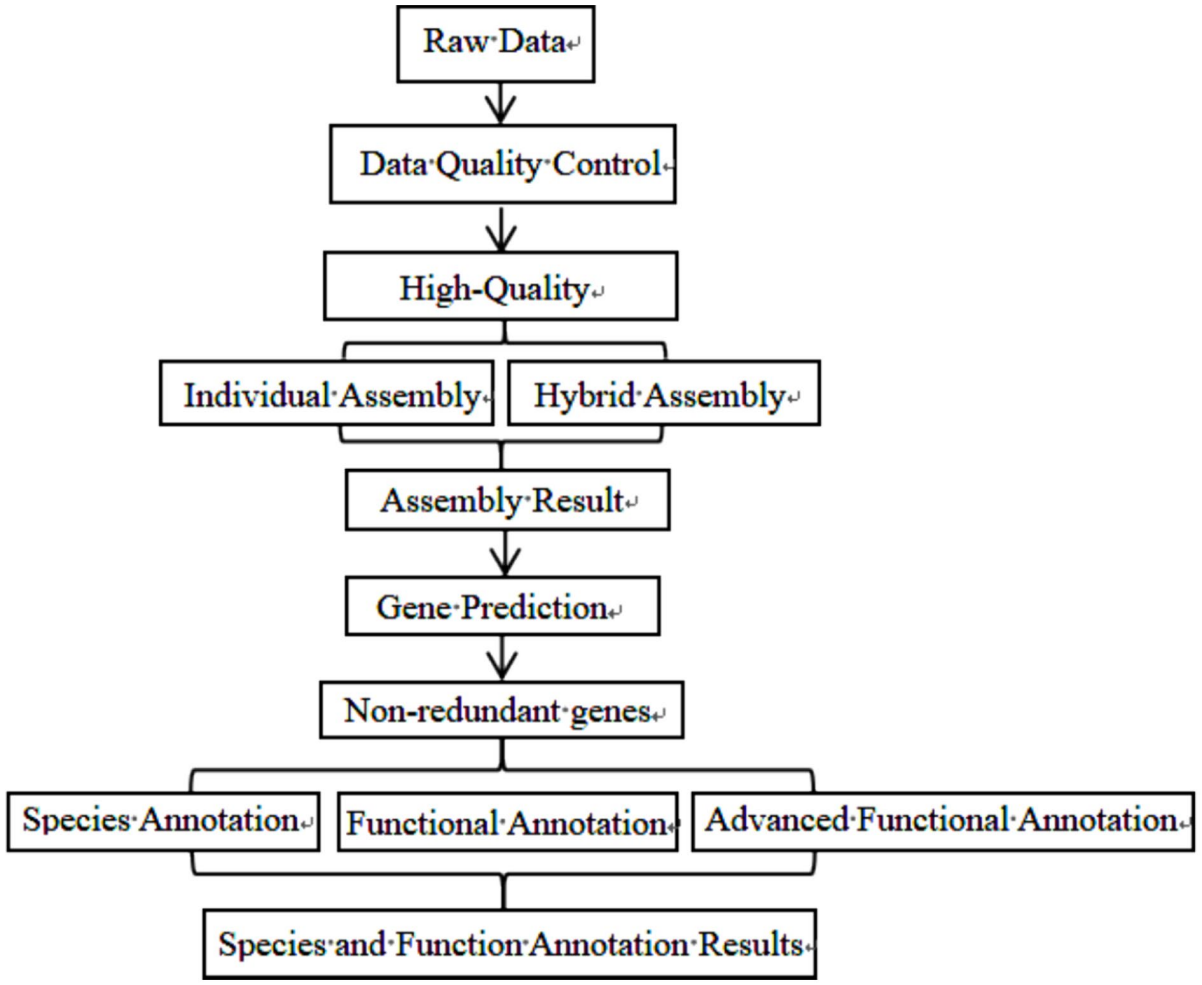
Information analysis flow chart.

After data quality control, Metagenome assembly, gene prediction, species annotation, and standard functional database annotation were performed. The specific biological information analysis process has been highlighted in Figure 2.

### 2.6 Data statistics and analysis

Sequences containing more than a certain proportion of low-quality bases (quality score ≤ 38) were removed (40 bp by default). Reads with a specific ratio of N-bases were also discarded (10 bp by default). Additionally, reads that overlapped with an adapter beyond a certain threshold were eliminated (15 bp by default). If host contamination was detected in the sample, it was compared with the host sequence to filter out reads potentially originating from the host. After preprocessing, clean data were obtained, and SOAP denovo assembly software was utilized for assembly analysis. Scaftigs generated by single sample and mixed assembly below 500 bp were filtered out. Statistical analysis and subsequent gene prediction were then performed on the remaining fragments. For each sample and scaftigs (≥ 500 bp), MetaGeneMark was used to predict open reading frames (ORFs). Any information shorter than 100 nt was excluded based on the expected results. Gene prediction involved analyzing assembled genome sequences to identify functional regions, such as genes in known organisms, based on knowledge of their genetic structure or database sequences. Genes were used to check against functional databases; DIAMOND software compared unigenes with sequences of bacteria, fungi, archaea, and viruses extracted from the National Center for Biotechnology Information database. The results of these comparisons were analyzed, and following filtering, the classification level before the first branch was used as the species annotation information for the sequence. Metagenomic species analysis included column and heat map analysis of species abundance and principal component analysis. The BLASTP function of the non-redundant gene application software Diamond was annotated. The diamond software compared unigenes with various functional databases, primarily using the KEGG (Kyoto Encyclopedia of Genes and Genomes) Database and the CAZy (Carbohydrate Active enzymes Database). Antibiotic resistance genes were annotated using The Comprehensive Antibiotic Resistance Database (CARD), and the abundance distribution of antibiotic resistance genes was obtained.

Finally, the significance of differences between groups was analyzed using SPSS 17.0 statistical software and expressed as mean ± standard deviation. Using a one-way analysis of variance, a *p*-value <0.05 was considered a significant difference, and a *p*-value <0.01 was considered an extremely significant difference.

## 3 Results

### 3.1 Effects of concentrate feeding sequence on volatile fatty acids of Dezhou donkey

The study investigated the influence of different concentrate feeding sequences on the VFA concentrations in Dezhou donkeys. Notably, valeric acid exhibited significant variations across the dietary treatments. The levels of valeric acid in both the *CF* and TMR groups were significantly higher than FC group (*p* < 0.05). However, there was no significant difference between the *CF* and TMR groups (*p* > 0.05) in valeric acid levels. Acetic acid shows a significant difference trend. In contrast, Total VFA, PH, propionic acid, butyric acid, and isovaleric acid did not show significant different among the treatments (*p* > 0.05; Table 3).

**Table 3:** Effects of feeding regimen on volatile fatty acids (μg/mL) of Dezhou donkey.

Items	Groups			*p*-value
	FC	*CF*	TMR	
PH	6.07 ± 0.66	6.36 ± 0.83	6.20 ± 0.63	0.819
Total VFA	103.33 ± 30.07	97.77 ± 29.25	121.14 ± 20.95	0.395
Acetic acid	63.00 ± 6.72	69.33 ± 6.68	84.81 ± 2.46	0.059
Propionic acid	25.87 ± 3.42	29.45 ± 3.33	30.51 ± 3.82	0.627
Butyric acid	6.85 ± 1.67	7.70 ± 1.53	9.59 ± 0.65	0.447
Valeric acid	0.34 ± 0.05^b^	0.55 ± 0.07^a^	0.67 ± 0.07^a^	0.018
Isovaleric acid	0.49 ± 0.08	0.51 ± 0.08	0.56 ± 0.06	0.829

The data are the means of five replicates per treatment (*n* = 5). The values with different letter superscripts mean significant differences (*p* < 0.05). VFA, volatile fatty acids.

### 3.2 Macrogenomic sequencing and assembly quality analysis of cecum microorganisms

**Figure 3:**
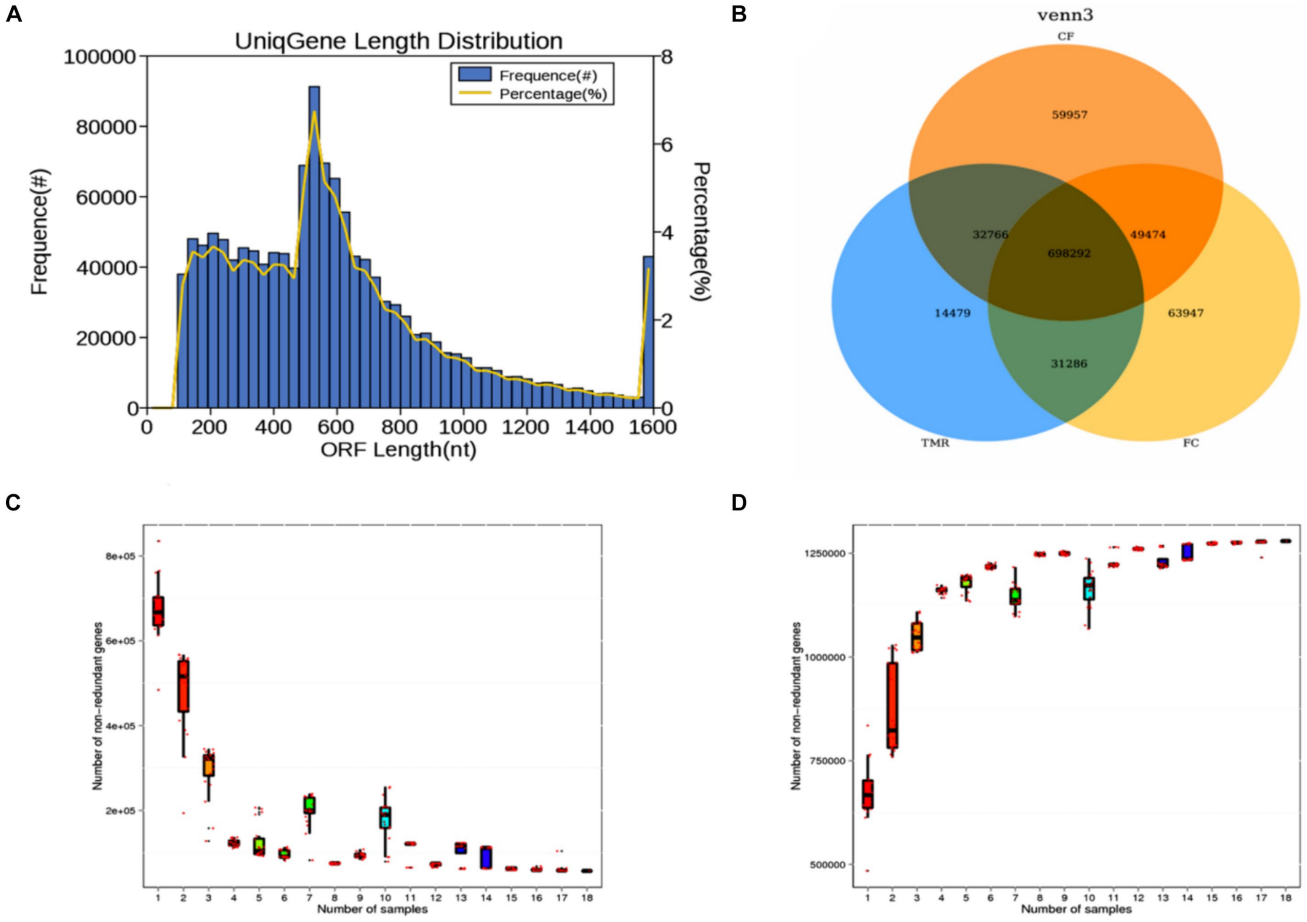
**(A)** Statistical diagram of length distribution of gene catalog. **(B)** Gene number Venn analysis graph. **(C)** Core gene dilution curve. **(D)** Pan gene dilution curve.

This investigation utilized metagenomic sequencing to analyze nine intestinal content samples. The length distribution of gene fragments in the sequenced samples predominantly ranged range from 100 to 1,600 nt. Notably, gene fragments measuring between 200 nt and 600 nt constituted the majority, while those of longer lengths were less prevalent (Figure 3A). A Venn diagram analysis highlighted distinct gene types across the three sample groups. The number of specific genes in TMR, *CF*, and FC groups were 14,479, 59,957, and 63,947, respectively. TMR and *CF* had a total of 32,766 genes. TMR and FC had a total of 31,286 genes. There were 49,474 genes in *CF* and group. The total number of genes in the three groups was 698,292 (Figure 3B). It can be seen from the gene dilution curve that the number of samples had sufficient species coverage. With the increase of sequencing samples, the number of genes gradually stabilized. Correlation analysis of gene expression levels among samples showed differences, indicating that the data were statistically significant and could represent the composition of intestinal microbial species in three different concentrate feeding sequences (Figures 3C,D).

### 3.3 Analysis of cecal microbial diversity across different concentrate feeding sequences

Species annotation and abundance analysis were performed on the metagenomic sequencing data of cecum microorganisms of three groups of experimental donkeys. At the gate level, the dominant phyla in the cecum of each group mainly included Bacteroidetes, Firmicutes, and Proteobacteria. However, there was little difference in the dominant microbial species at the gate level. Bacteroidetes accounted for the most significant proportion, about 50%. The feeding sequence of concentrate had a substantial impact on Firmicutes, Lentisphaerae, and Verrucomicrobia (*p* < 0.05), indicating that the *CF* group was significantly higher than the FC group and TMR group (*p* < 0.05; Table 4). On a subordinate level, analysis of the top 10 cecal microbiota in fattening donkeys with different precision feeding orders. The dominant bacteria genera in cecum mainly include *Prevotella*, *Bacteroides*, and *Fibrobacter*. There was little difference in the dominant microbial species at the generic level. *Clostridium* in the feeding sequence had a significant effect (*p* < 0.05), and the FC group was significantly higher than the *CF* group and TMR group (*p* < 0.05; Table 5).

**Table 4:** *p*-Values of relative abundance of species at the phylum levels.

Phyla	Groups			*p*-value
	FC	*CF*	TMR	
Bacteroidetes	0.50502 ± 0.01706	0.51090 ± 0.00375	0.53921 ± 0.01128	0.246
Firmicutes	0.11448 ± 0.00021^a^	0.19748 ± 0.02228^b^	0.12553 ± 0.00152^a^	0.036
Proteobacteria	0.01323 ± 0.00252	0.02096 ± 0.00243	0.02099 ± 0.00291	0.200
Fibrobacteres	0.01155 ± 0.00183	0.01324 ± 0.00173	0.01117 ± 0.00028	0.618
Euryarchaeota	0.00037 ± 0.00004	0.00033 ± 0.00002	0.00031 ± 0.00001	0.387
Spirochaetes	0.01854 ± 0.00006	0.02325 ± 0.00011	0.01901 ± 0.00437	0.449
Lentisphaerae	0.00106 ± 0.00004^ab^	0.00141 ± 0.00017^b^	0.00065 ± 0.00001^a^	0.028
Chytridiomycota	0.00268 ± 0.00048	0.00237 ± 0.00030	0.00181 ± 0.00065	0.514
Verrucomicrobia	0.00141 ± 0.00003^b^	0.00171 ± 0.00006^c^	0.00103 ± 0.00006^a^	0.007
Ascomycota	0.00180 ± 0.00041	0.00112 ± 0.00026	0.00183 ± 0.00016	0.305

The data are the means of five replicates per treatment (*n* = 5). The values with different letter superscripts mean significant differences (*p* < 0.05).

**Table 5:** *p*-Values of relative abundance of species at the genus levels.

Phyla	Groups			*p*-value
	FC	*CF*	TMR	
Prevotella	0.21604 ± 0.00887	0.20535 ± 0.00528	0.21968 ± 0.00435	0.319
Bacteroides	0.07701 ± 0.00108	0.08277 ± 0.00393	0.08844 ± 0.00252	0.133
Streptococcus	0.00215 ± 0.00134	0.00141 ± 0.00006	0.00121 ± 0.00063	0.741
Fibrobacter	0.01289 ± 0.00177	0.01448 ± 0.00153	0.01095 ± 0.00028	0.323
Treponema	0.02261 ± 0.00007	0.01782 ± 0.00004	0.01845 ± 0.00432	0.442
Campylobacter	0.00051 ± 0.00006	0.00161 ± 0.00050	0.00113 ± 0.00027	0.217
Methanocorpusculum	0.00013 ± 0.00002	0.00005 ± 0.00004	0.00001 ± 0.00001	0.562
Clostridium	0.00761 ± 0.00031^b^	0.00579 ± 0.00002^a^	0.00638 ± 0.00011^a^	0.014
Alistipes	0.00977 ± 0.00011	0.00978 ± 0.00105	0.00757 ± 0.00009	0.132
Anaerovibrio	0.01028 ± 0.00224	0.00427 ± 0.00111	0.00325 ± 0.00014	0.076

The data are the means of five replicates per treatment (*n* = 5). The values with different letter superscripts mean significant differences (*p* < 0.05).

**Figure 4:**
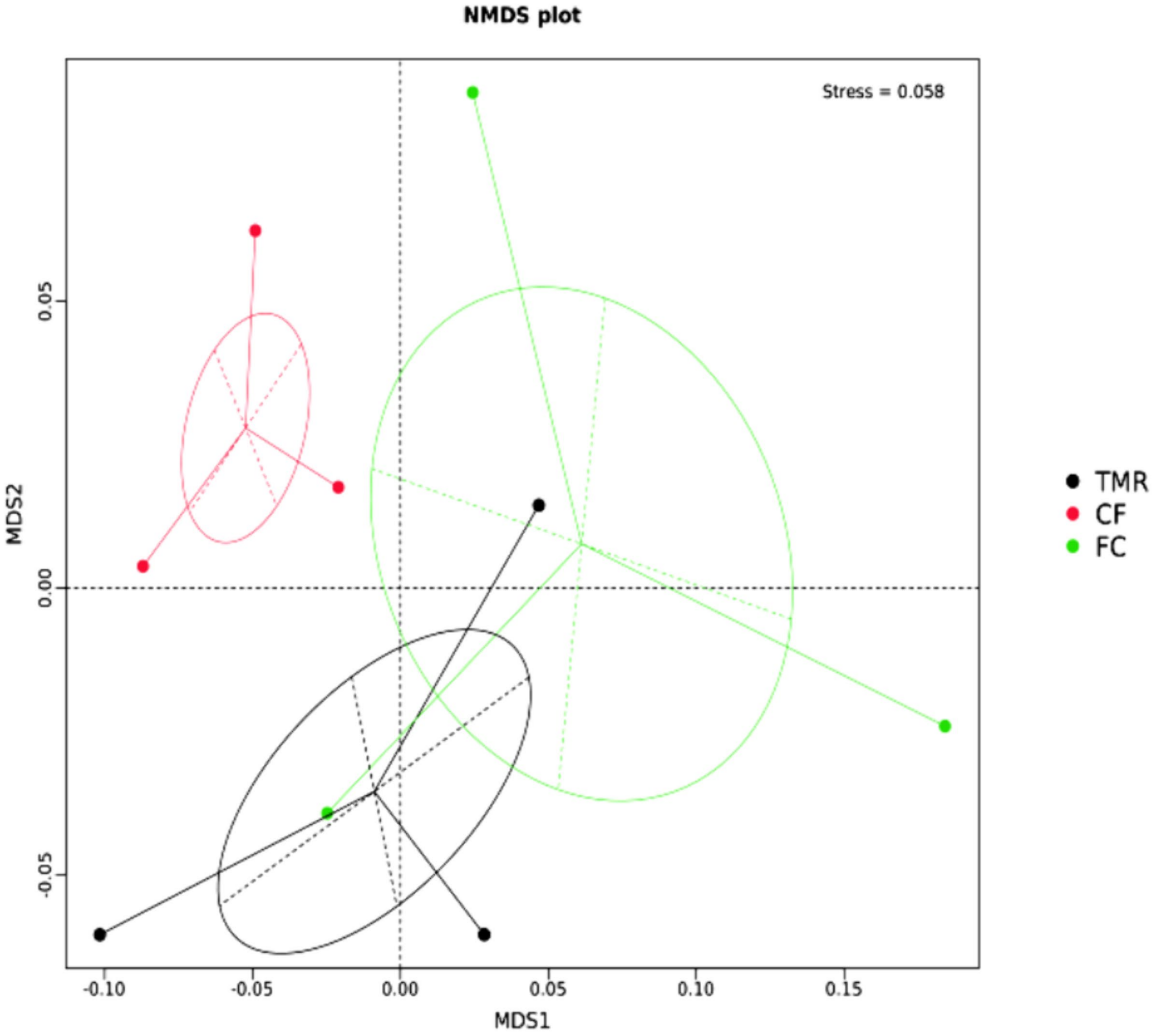
NMDS analysis at gate level.

The β diversity of each sample was analyzed and measured by Non-metric Multidimensional Scaling, Stess = 0.058 < 0.2, indicating that the analysis was reliable (Figure 4). The microbe community of cecum was scattered significantly in all groups, and the β diversity of *CF* differed from that of FC and TMR.

### 3.4 Metagenomic functional annotation and analysis

#### 3.4.1 Gene set KEGG annotation results

**Figure 5:**
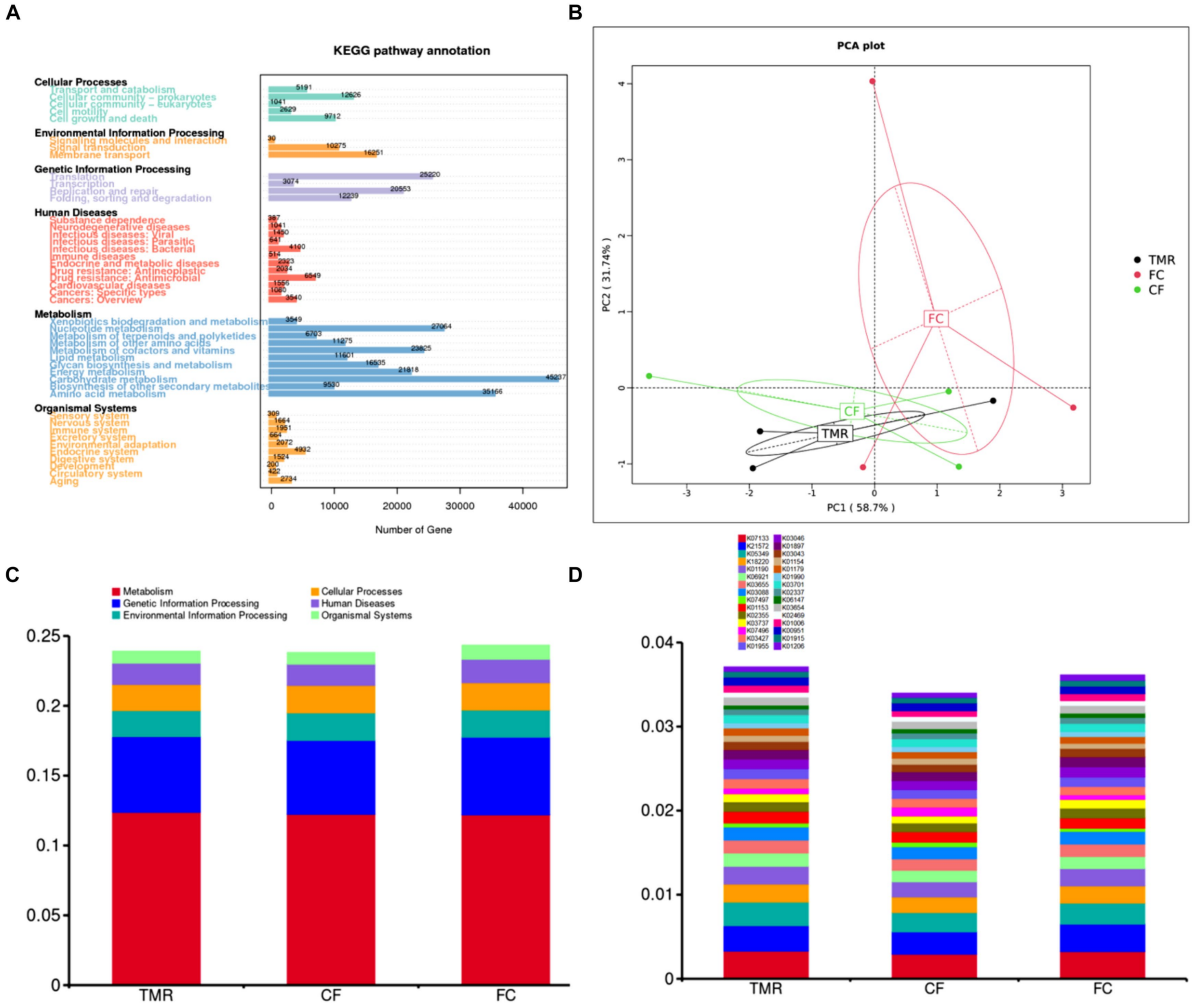
**(A)** Statistics of the number of unigenes annotations in KEGG. **(B)** KEGG’s gate level-based PCA results display chart for species. **(C)** KEGG’s level 1 function annotation relative abundance bar chart. **(D)** KEGG’s KO functional abundance relative abundance column chart.

In the KEGG PATHWAY database, biological metabolic pathways are divided into 6 categories, each of which is systematically classified into two, three, and four layers. Carbohydrate metabolism has the most significant number of annotated genes, followed by Amino acid metabolism and Nucleotide metabolism. All three are secondary metabolic functions under the KEGG primary function Metabolism classification. The KEGG three-level functional classification of the Global and Overview Atlas includes Metabolic pathways, which contain the primary metabolic functions or processes necessary for survival and thus have the highest number of genes analyzed in most studies (Figure 5A). Through PCA analysis under KEGG, it can be found that the interpretation degree of horizontal coordinate (PC1) on the difference effect is 58.7%, and that of vertical coordinate (PC2) on the difference effect is 31.7%. The feeding sequence of the three groups of different concentrates is far apart, and the functional similarity of *CF* and TMR is high, while that of FC is low (Figure 5B). According to the annotation results of the top 10 relative abundance, Metabolism and Genetic Information Processing are the two main biological metabolic pathways with high abundance (Figure 5C). The six functional classes had no significant difference (*p* < 0.05). As depicted in the bar chart of the top 30 KOs based on relative abundance, TMR is annotated with the highest total number of genes, whereas *CF* is annotated to the lowest whole number of genes (Figure 5D). *CF* is significantly lower than FC and TMR in K01190, K03737, and K01006 (*p* < 0.05; Table 6). Query from KEGG database, K01190, K03737, and K01006 correspond to *lacZ* gene, *Por/nifJ* gene, and *ppdK* gene. The *lacZ* gene (K01190) encodes Beta-galactosidase, which primarily affects the galactose metabolic aspect of carbohydrates. The *Por/nifJ* gene (K03737) encodes Pyruvate Ferredoxin/Flavodoxin Oxidoreductase, which mainly affects the glycolysis aspects of carbohydrates. The *ppdK* (K01006) gene encodes Pyruvate Orthophosphate Dikinase, which primarily affects the glycolysis aspects of carbohydrates. It is concluded that galactose metabolism and glycolysis are more active when fed a TMR or fiber diet followed by a concentrate diet.

**Table 6:** *p*-Values of the top 30 relative abundances of functional genes at the KO level.

Genes	Groups			*p*-value
	*CF*	FC	TMR	
K07133	0.00293 ± 0.00012	0.00325 ± 0.00007	0.00331 ± 0.00015	0.134
K21572	0.00264 ± 0.00023	0.00324 ± 0.00025	0.00300 ± 0.00010	0.186
K05349	0.00232 ± 0.00017	0.00252 ± 0.00017	0.00283 ± 0.00006	0.111
K18220	0.00184 ± 0.00009	0.00204 ± 0.00007	0.00212 ± 0.00012	0.184
K01190	0.00181 ± 0.00005^a^	0.00208 ± 0.00010^b^	0.00213 ± 0.00005^b^	0.040
K06921	0.00136 ± 0.00012	0.00144 ± 0.00003	0.00157 ± 0.00008	0.281
K03655	0.00137 ± 0.00006	0.00148 ± 0.00007	0.00154 ± 0.00006	0.251
K03088	0.00143 ± 0.00007	0.00151 ± 0.00007	0.00156 ± 0.00003	0.376
K07497	0.00055 ± 0.00017	0.00038 ± 0.00004	0.00047 ± 0.00007	0.566
K01153	0.00122 ± 0.00007	0.00120 ± 0.00002	0.00136 ± 0.00005	0.123
K02355	0.00106 ± 0.00005	0.00120 ± 0.00007	0.00117 ± 0.00004	0.253
K03737	0.00082 ± 0.00004^a^	0.00103 ± 0.00006^b^	0.00095 ± 0.00002^ab^	0.047
K07496	0.00103 ± 0.00024	0.00054 ± 0.00003	0.00065 ± 0.00011	0.129
K03427	0.00105 ± 0.00003	0.00100 ± 0.00003	0.00113 ± 0.00004	0.099
K01955	0.00108 ± 0.00007	0.00113 ± 0.00004	0.00122 ± 0.00005	0.276
K03046	0.00011 ± 0.00076	0.00007 ± 0.00104	0.00007 ± 0.00096	0.138
K01897	0.00104 ± 0.00004	0.00117 ± 0.00008	0.00111 ± 0.00003	0.313
K03043	0.00090 ± 0.00005	0.00102 ± 0.00006	0.00099 ± 0.00003	0.234
K01154	0.00072 ± 0.00002	0.00060 ± 0.00004	0.00073 ± 0.00003	0.067
K01179	0.00077 ± 0.00004	0.00080 ± 0.00005	0.00087 ± 0.00004	0.352
K01990	0.00061 ± 0.00005	0.00059 ± 0.00005	0.00061 ± 0.00003	0.916
K03701	0.00093 ± 0.00002	0.00098 ± 0.00006	0.00096 ± 0.00001	0.602
K02337	0.00071 ± 0.00001	0.00072 ± 0.00005	0.00070 ± 0.00001	0.842
K06147	0.00048 ± 0.00002	0.00049 ± 0.00004	0.00044 ± 0.00003	0.547
K03654	0.00090 ± 0.00001	0.00093 ± 0.00005	0.00097 ± 0.00002	0.269
K02469	0.00057 ± 0.00002	0.00057 ± 0.00004	0.00057 ± 0.00001	0.983
K01006	0.00068 ± 0.00003^a^	0.00083 ± 0.00003^b^	0.00084 ± 0.00003^b^	0.019
K00951	0.00090 ± 0.00003	0.00088 ± 0.00006	0.00094 ± 0.00001	0.524
K01915	0.00067 ± 0.00003	0.00070 ± 0.00002	0.00070 ± 0.00001	0.592
K01206	0.00050 ± 0.00004	0.00064 ± 0.00005	0.00051 ± 0.00003	0.111

The data are the means of five replicates per treatment (*n* = 5). The values with different letter superscripts mean significant differences (*p* < 0.05).

#### 3.4.2 Gene set CAZy annotation results

**Figure 6:**
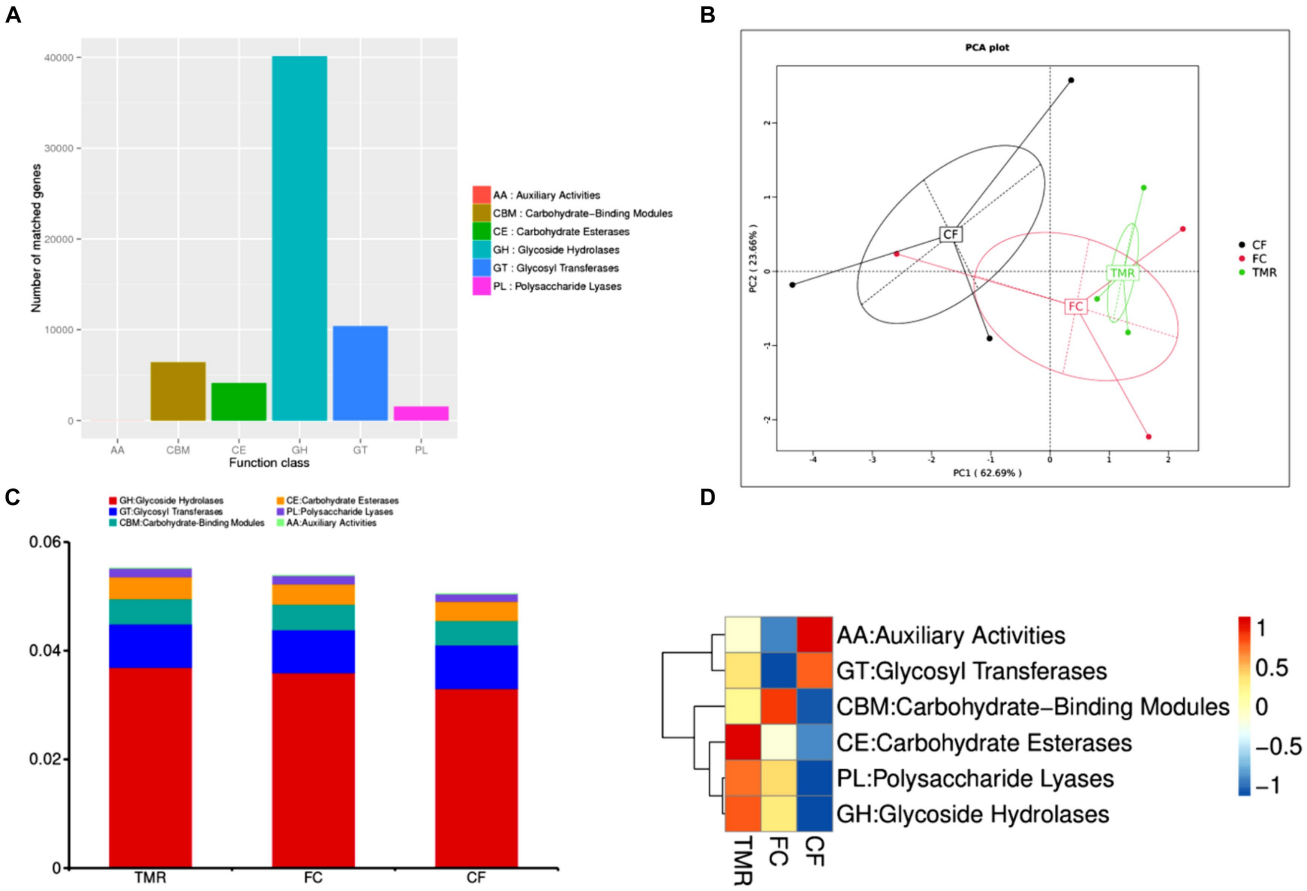
**(A)** Statistics of the number of Unigenes annotations in CAZy. **(B)** Display of PCA results for CAZy. **(C)** CAZy’s level 1 function annotation relative abundance bar chart. **(D)** Functional abundance clustering heatmap of CAZy.

The CAZy database mainly covers six functional categories. Among these, glycoside hydrolases are the most frequently annotated function, indicating that they are the predominant enzyme system in the cecal microorganisms for carbohydrate metabolism (Figure 6A). Through PCA analysis, it can be seen that the explanation degree of horizontal coordinate (PC1) for the difference influence is 62.69%, and that of vertical coordinate (PC2) for the difference influence is 23.66%. The distance of intestinal flora in the three different concentrate feeding sequences was far, indicating that the functional composition of intestinal flora was less similar (Figure 6B). According to the annotation results of the top 10 relative abundances, Glycoside Hydrolases (GHs) and Glycosyl Transferases (GTs) have a high functional abundance (Figure 6C). The functional abundance of *CF* cecum microflora was significantly different from that of the other two groups (Figure 6D).

#### 3.4.3 Analysis of antibiotic resistance genes annotation results

**Figure 7:**
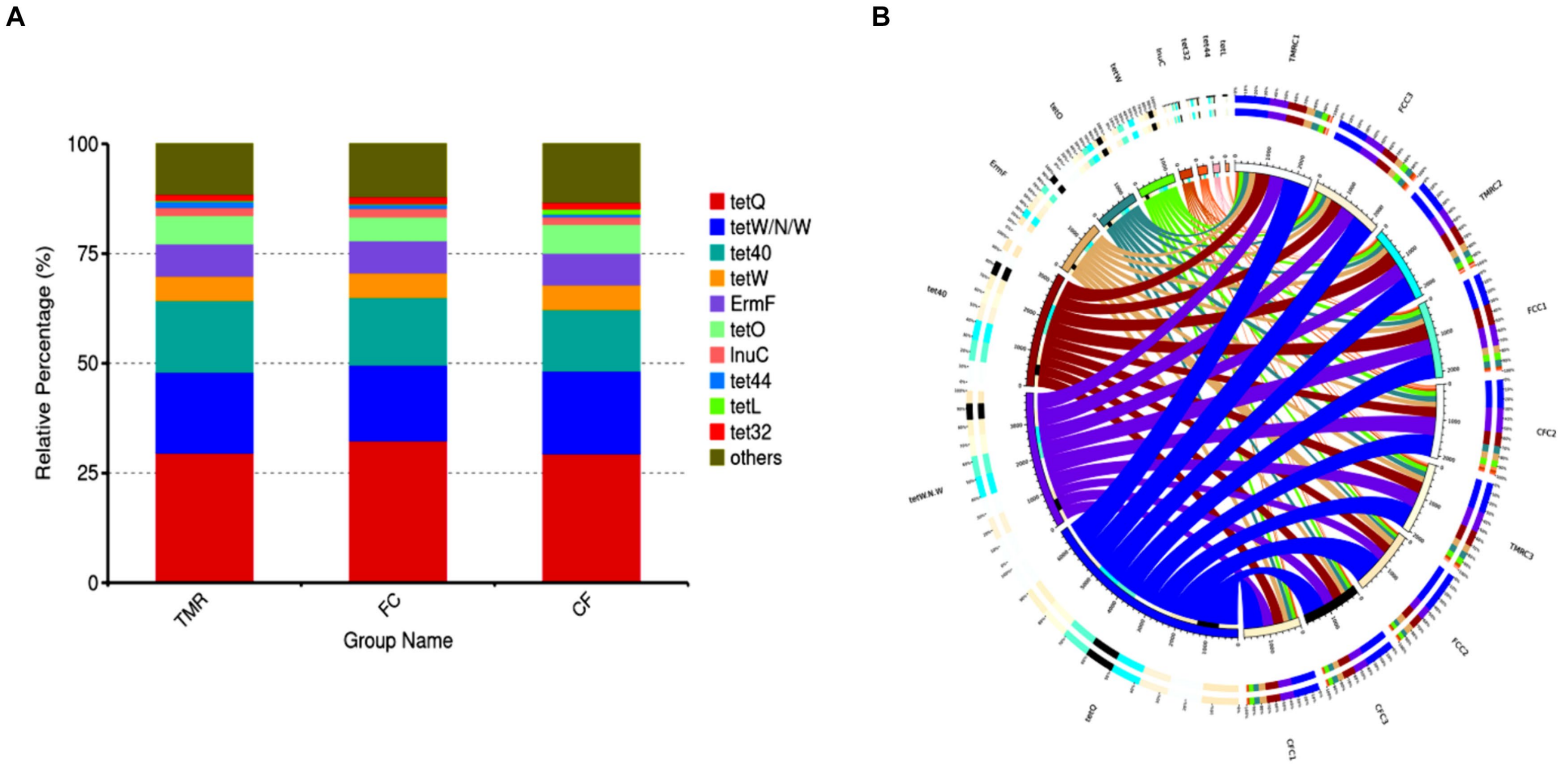
**(A)** Abundance bar chart of different AROs in each group. **(B)** Overview of antibiotic resistance genes.

**Figure 8:**
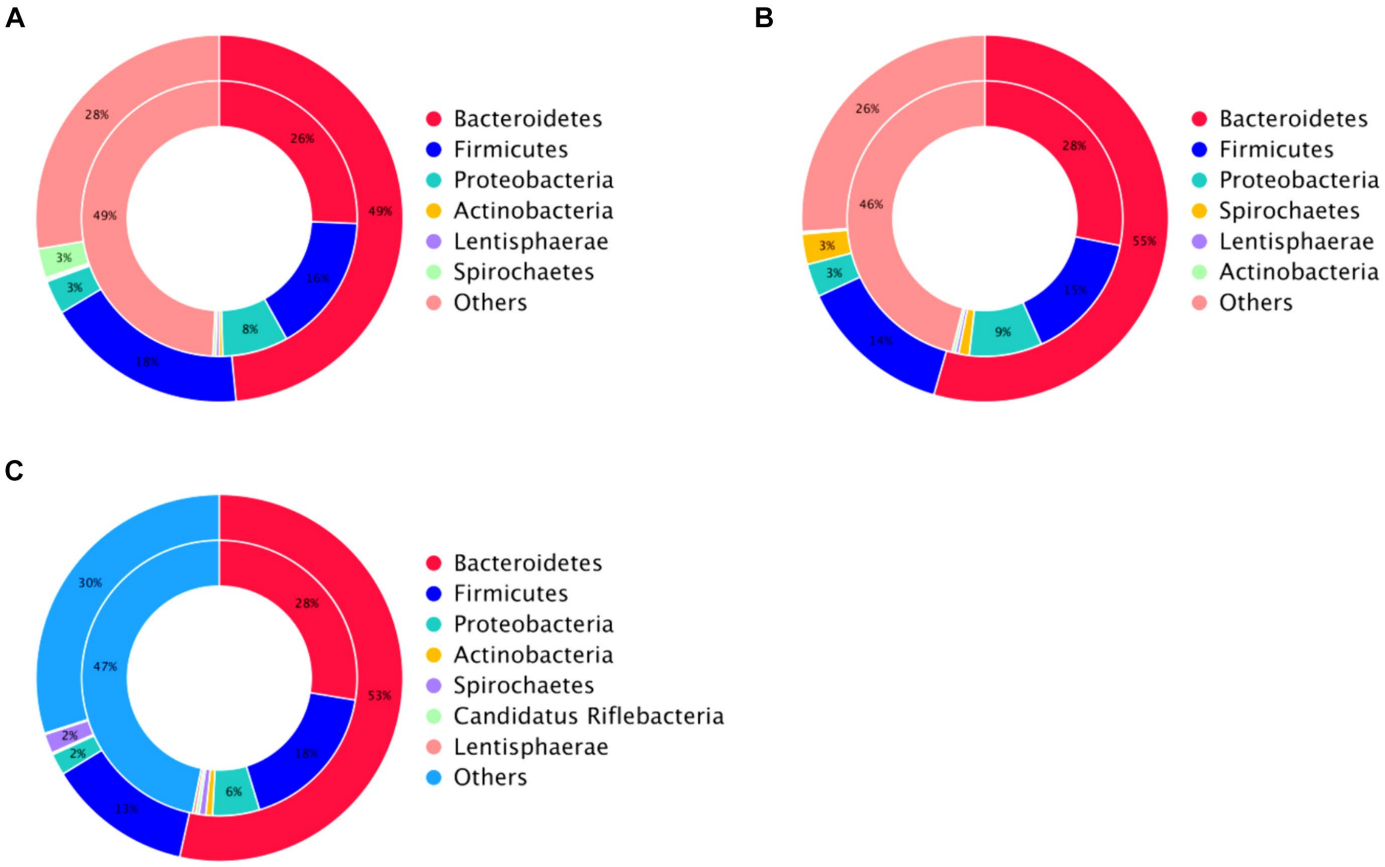
Analysis circle of resistance gene species attribution. **(A)** Analysis circle of *CF* resistance gene species attribution. **(B)** TMR resistance gene species attribution analysis circle. **(C)** Circle diagram of FC resistance gene species attribution analysis.

Antibiotic resistance genes are present in the gut microbiota of both human and animal. Based on the CARD database, this study started from the top 10 relative abundance of antibiotic resistance genes (Figure 7). Notably, the contents of antibiotic resistance genes (*tetQ*, *tetW/N/W* and *tet40*) were higher in cecal microorganisms. Conversely, the levels of *tetO*, and *tet44* in the *CF* were significantly higher compared to those in the FC and TMR (*p* < 0.05; Table 7). The *tetO* gene is known to encode ribosome protection proteins that bind to ribosomes and induce alterations in their structural configuration. Species annotation of all sample groups facilitated the acquisition of specific species information corresponding to each antibiotic resistance gene. Comparative analysis based on the known species associated with these genes intensely demonstrates a higher prevalence of antibiotic resistance genes within the phyla Bacteroidetes, Firmicutes, and Proteobacteria (Figure 8).

**Table 7:** The *p*-value of the relative abundance of different AROs in each group.

AROs	Groups			*p*-value
	FC	*CF*	TMR	
tetQ	28.5350 ± 0.1250	31.6000 ± 1.8500	29.5500 ± 0.6424	0.237
tetW/N/W	19.2250 ± 0.4150	16.6100 ± 0.0100	18.4000 ± 0.6071	0.068
tet40	13.6850 ± 1.2550	14.9950 ± 0.6650	17.2200 ± 0.3500	0.126
tetW	6.2000 ± 0.0400	5.5767 ± 0.2224	5.5333 ± 0.2822	0.228
ErmF	6.7600 ± 0.0500	6.7600 ± 0.3800	7.2350 ± 0.0150	0.348
tetO	5.8350 ± 0.0750^a^	6.2950 ± 0.0150^b^	6.7600 ± 0.0300c	0.002
lnuC	1.8650 ± 0.0250	1.8300 ± 0.1000	1.9750 ± 0.0450	0.38
tet44	0.9900 ± 0.0300^a^	0.7900 ± 0.0100^a^	1.4800 ± 0.0300^b^	0.001
tetL	0.0950 ± 0.0350	0.1700 ± 0.0800	0.0450 ± 0.0150	0.351
tet32	1.2250 ± 0.1350	1.2250 ± 0.0550	1.3850 ± 0.0250	0.421

The data are the means of five replicates per treatment (*n* = 5). The values with different letter superscripts mean significant differences (*p* < 0.05).

## 4 Discussion

Nutrient digestibility is an important index to evaluate the nutritional value of diets, reflecting the digestion and absorption status of nutrients, and also reflecting the nutritional balance of diets (24). In the current study, we evaluated the nutrient digestibility of various concentrate feeding sequences. Our findings revealed that the digestibility of DM and ADF in the *CF* feeding sequence was superior compared to other sequences, with statistically significant differences observed. These results suggest that inconsistencies in concentrate feeding sequences can significantly influence the effectiveness of livestock nutrition. Specifically, when concentrate feeds, which are known for their palatability and strong appetitive responses, are administered first, they enhance nutrient digestion and absorption, thereby improving overall nutrient digestibility (14). Conversely, the consumption of roughage, characterized by its satiating properties, may limit overall feed intake and subsequently nutrient uptake.

The microbial fermentation of feed with in the digestive tract leads to the production of substantial quantities of VFAs, which serve as critical sources of energy and nutrients for herbivores (25). Among these, acetic acid plays a pivotal role in animal energy metabolism, acting as a precursor for the synthesis of both milk fat and body fat (26). Propionic acid, another significant VFA, is instrumental in facilitating the transport and storage of glucose (27). In this study, no significant differences were observed between propionic acid and butyric acid concentrations; however, acetic acid exhibited a significant difference trend. Additionally, the concentration of valerate in the FC feeding sequence was significantly lower than the other two feeding sequences examined. Our findings further indicate that the inclusion of concentrate in the diet, compared to a fiber-only diet, alters the VFA concentration in the cecum (28).

Horses, as non-ruminant herbivores, demonstrate a relatively weak capacity for starch digestion in the small intestine. Excessive starch intake, surpassing the small intestine’s digestive capability, leads to substantial fermentation in the hindgut. This fermentation produces acids that lower the pH of the intestine, thereby impacting the digestibility of feed nutrients and potentially endangering the balance of the hindgut microbial ecosystem (29). Comparative studies have revealed that different concentrate feeding sequences significantly affect animal weight and daily gain (30). Specifically, experiments evaluating various sequences of concentrate and raw feed intake found that the digestibility of raw feed following concentrate consumption was significantly higher than when fed after unconcentrated feed, enhancing feed efficiency (31). The TMR when compared with separate feeding of concentrate and fiber, not only increase feed intake but also enhance weight gain and growth rate, yielding favorable economic benefits (32). In equine studies, feeding concentrate feed before fiber significantly increased average daily gain and final weight compared to other feeding methods, improved nutrient digestibility, and enhanced protein absorption, resulting in better growth performance in horses (33). This experimental study demonstrates that the *CF* sequence achieves superior growth performance and daily gain. The results of this experiment, however, are not entirely consistent with those from studies involving dairy ruminants fed TMR, likely due to the differing impacts on the stability of the rumen environment and the promotion of fermentation. This discrepancy can be attributed to the unique characteristics of equine digestion, which primarily occurs via hindgut fermentation.

The gastrointestinal tract of animals hosts a diverse and abundant microbial community, crucial for regulating various physiological functions including the digestion and absorption of nutrients (34). Numerous factors influence the diversity of intestinal microbes, particularly in equines like horses and donkeys, with age, diet, and other factors playing significant roles (35). In the context of dietary energy gain, diet is a predominant factor affecting the cecal microflora and fermentation parameters in equines (36).

This study investigated the impact of altering the feeding sequence of concentrates on the cecal microorganisms in equines. The experiment identified Bacteroidetes and Firmicutes as the dominant bacterial phyla, with a significantly higher abundance of Firmicutes observed in the *CF* group compared to the FC and TMR groups (*p* < 0.05). Bacteroides play a critical role in the metabolism of carbohydrates and other nutrients, contributing to the maintenance of normal intestinal functions. Firmicutes, known for producing short-chain fatty acids (37), predominantly consist of beneficial, spore-forming bacteria capable of withstanding dehydration and extreme environments, which also enhances energy extraction from food (38). In this experiment, there were differences in the cecal microbial structure of three types of Dezhou donkeys with different feeding sequences, indicating that varying the sequence of concentrate feeding could change the composition of cecal microorganisms.

Advancements in high-throughput sequencing and data analysis technology, have enabled the application of 16S rRNA gene amplicon sequencing to detect bacterial populations in complex samples like feces and colon regions, uncovering many previously unidentified bacteria (39). Metagenomic sequencing has been utilized to analyze human intestinal flora, revealing key pathways involved in polysaccharide and amino acid metabolism (40). These are crucial for studying the complex microbial communities in samples with diverse microorganisms. However, when applied to complex microbiological samples, metagenomic sequencing is an invaluable tool for microbial identification and functional analysis (41). Analysis of the cecal microbiota in fattening donkeys using CAZy annotation revealed that glycoside hydrolase was the predominant enzyme system involved in carbohydrate metabolism. KEGG pathway enrichment analysis further identified carbohydrate metabolism as the principal metabolic pathway in the cecal microflora, followed by amino acid and nucleotide metabolism. Through one-way ANOVA, there was no significant difference in the six functional categories. In the KEGG ORTHOLOGY database, the TMR group was annotated to the highest total number of genes, and the *CF* group was annotated to the lowest total number of genes. Through one-way ANOVA, *CF* group of K01190, K03737 and K01006 channels was significantly lower than that of FC group and TMR group. According to the gene information in KEGG database, *lacZ*, *Por/nifJ* and *ppdK* genes of K01190, K03737 and K01006 pathways mainly regulate the galactose metabolism of carbohydrates and glycolysis of carbohydrates. It is concluded that galactose metabolism and glycolysis are more active when feeding TMR diet or roughage feed first and then concentrate feed, and the utilization rate of *CF* to crude fiber is lower.

This experiment demonstrated that altering the feeding order of concentrate impacts the cecal microbiota of adult fattening donkeys. Specifically, when concentrate was fed first, there was an observed increase in beneficial bacteria such as Bacteroidetes and Firmicutes. These groups are pivotal in carbohydrate and other nutrient metabolism, contributing to the maintenance of normal intestinal physiology Notably, many Firmicutes enhance energy extraction from feed, thereby potentially improving growth performance. Metagenomic analysis suggests that these effects may be attributed to the microbes’ reduced capacity to utilize crude fiber. It is important to note that this study was limited to cecal microbiota samples from the production process of fattening donkeys, and did not account for environmental variations that could affect functional outcomes.

The study also addressed the widespread presence of antibiotic resistance genes in both human and environmental microbiomes, exacerbated by antibiotic overuse which leads to irreversible changes in microbial communities and environments. This poses significant risks to human health and ecological stability (42). Utilizing the CARD resistance gene database, our research identified higher levels of antibiotic resistance genes in Bacteroides, Firmicutes and Proteobacteria. Notably, the contents of *tetO* and *tet44* were significantly higher in the *CF* group compared to the TMR and FC groups, suggesting enhanced drug resistance in the *CF* group. The underlying reasons for these differences warrant further investigation. These findings provide insights into the role of gut flora in complex traits, providing direction for the next step in feeding.

## 5 Conclusion

In conclusion, this study employed metagenomic technology to elucidate the composition and functional dynamics of cecum microorganisms in Dezhou fattening donkeys. The analysis revealed Bacteroides and *Prevotella* as the predominant bacteria across all treated groups. While the structural composition of the cecum flora and primary metabolic pathways remained consistent among groups, notable differences were observed in functional performance between *CF* and the other two groups. Specifically, the *CF* group exhibited significantly reduced abundance of *lacZ*, *Por/nifJ*, and *ppdK* gene, which are critical in galactose metabolism and glycolysis, indicating a lower efficiency in coarse fiber utilization compared to the FC and TMR groups. Additionally, the *CF* group displayed a higher contents of intestinal flora antibiotic resistance genes, *tetO* and *tet44,* particularly within the Bacteroidetes, Firmicutes, and Proteobacteria, suggesting enhanced microbial resistance. Altogether, these findings suggest a sequential feeding regimen, initiating with fine feed followed by fiber feed, to optimize the growth performance and economic benefits of Dezhou fattening donkeys in agricultural practices.

## Data availability statement

The data presented in the study are deposited in the National Center for Biotechnology Information repository, accession number PRJNA1116530.

## Ethics statement

The animal study was approved by Special Committee of research ethics, Liaocheng University. The study was conducted in accordance with the local legislation and institutional requirements.

## Author contributions

CC: Writing – original draft, Writing – review & editing. LX: Data curation, Writing – review & editing. JX: Data curation, Writing – review & editing. TL: Data curation, Writing – review & editing. XQ: Data curation, Writing – review & editing. LL: Investigation, Writing – review & editing. XC: Investigation, Writing – review & editing. MA: Investigation, Writing – review & editing. YJ: Investigation, Supervision, Writing – review & editing. GL: Investigation, Supervision, Writing – review & editing.
